# Introducing Membrane Charge and Membrane Potential to T Cell Signaling

**DOI:** 10.3389/fimmu.2017.01513

**Published:** 2017-11-09

**Authors:** Yuanqing Ma, Kate Poole, Jesse Goyette, Katharina Gaus

**Affiliations:** ^1^EMBL Australia Node in Single Molecule Science, School of Medical Sciences, University of New South Wales, Sydney, NSW, Australia; ^2^ARC Centre of Excellence in Advanced Molecular Imaging, University of New South Wales, Sydney, NSW, Australia

**Keywords:** membrane lipids, T cell receptor signaling, zeta potential, CD3 zeta chain, transmembrane potential

## Abstract

While membrane models now include the heterogeneous distribution of lipids, the impact of membrane charges on regulating the association of proteins with the plasma membrane is often overlooked. Charged lipids are asymmetrically distributed between the two leaflets of the plasma membrane, resulting in the inner leaflet being negatively charged and a surface potential that attracts and binds positively charged ions, proteins, and peptide motifs. These interactions not only create a transmembrane potential but they can also facilitate the formation of charged membrane domains. Here, we reference fields outside of immunology in which consequences of membrane charge are better characterized to highlight important mechanisms. We then focus on T cell receptor (TCR) signaling, reviewing the evidence that membrane charges and membrane-associated calcium regulate phosphorylation of the TCR–CD3 complex and discuss how the immunological synapse exhibits distinct patterns of membrane charge distribution. We propose that charged lipids, ions in solution, and transient protein interactions form a dynamic equilibrium during T cell activation.

## Introduction

There are many reviews of membrane order/lipid rafts and how this property of the plasma membrane impacts T cell receptor (TCR) signaling [see Ref. (1, 2) for recent examples], but the importance of membrane charge to TCR signaling is only recently becoming appreciated. The purpose of this review is to act as a primer to the field of membrane charge for those who are interested in how it applies to TCR signaling. Since these biophysical concepts have not traditionally been associated with the immunology, we begin by exploring relevant concepts from electrophysiology and membrane biophysics to provide a context for recent advances in our understanding of TCR signaling.

The cell plasma membrane is composed of two layers of phospholipids with the hydrophilic head groups facing the aqueous intra- and extracellular environments, while the hydrophobic acyl chain aligns laterally forming the hydrophobic core of the bilayer. Not only does the plasma membrane act as the primary barrier to separate the cell from the external environment, but it is also the interface where many transmembrane signal transduction events occur. This is mainly conveyed through transmembrane proteins and peripheral membrane proteins that associate with the inner leaflet of the plasma membrane. A hallmark of transmembrane signaling, including TCR signaling, is that signaling reactions are (i) highly specific; for example, being only initiated by antigens, (ii) highly sensitive so that engagement of a few receptors is sufficient to trigger activation responses, and (iii) must be highly coordinated to prevent basal signaling in the absence of ligands. While most of the attention and focus has been given to the structure and conformational change of membrane proteins, it has become increasingly clear that the composition and distribution of the membrane lipids can affect the conformation and function of membrane proteins.

Early models of cell membranes simply depicted membrane lipids as fluid entities within a homogenous matrix, with their main function being the accommodation of membrane proteins. More recent models include the heterogeneous distribution of lipids both between the two leaflets and laterally within the membrane. Most phospholipids have an asymmetrical distribution between the outer and inner leaflets of the cell plasma membrane. While neutral phospholipids such as sphingomyelin and zwitterionic phosphatidylcholine are located primarily in the outer leaflet of the plasma membrane, most anionic phospholipids, such as phosphatidic acid (PA), phosphatidylserine (PS), phosphatidylethanolamine (PE), and phosphatidylinositol (PI) species, such as phosphatidylinositol 4,5-bisphosphate (PIP_2_) and phosphatidylinositol (3,4,5)-trisphosphate (PIP_3_) are mostly located at the inner leaflet (3). The low acid dissociation constant (pKa) values of the phosphate groups of the lipid head group are responsible for the negative charge of these lipids at physiological pH (3).

## The Plasma Membrane has Two Differently Charged Leaflets

The asymmetrical distribution of the phospholipids, particularly PS, has several biological impacts and it is highly conserved across eukaryotic cells (4, 5). While the lateral diffusion of the lipids within the monolayer is thermodynamically favorable, the transmembrane translocation of lipids between the two leaflets is thermodynamically challenging and, thus, mostly an adenosine triphosphate (ATP)-dependent process (5). The asymmetrical distribution of PS is established and maintained by flippase and floppase enzymes that move the lipids in and out of the two leaflets in opposite directions. The Ca^2+^-dependent scramblase moves the lipids in a bidirectional manner that counterbalances the asymmetrical distribution of lipids (5). The asymmetric arrangement of PS provides greater membrane mechanical stability through interactions of lipids within the cytosolic leaflet with the subjacent cytoskeletal proteins (4). In addition, the higher concentration of the conical shaped PS can induce negative curvature of the cell membrane (6). The disruption of lipid asymmetry has direct biological consequences. For instance, PS exposure is a mediator of blood coagulation in platelets and an activator of the scavenger receptors on macrophages for apoptosis (7). A deficit in TMEM16F scramblase expression leads to defects in PS translocation to the outer leaflet and impaired blood clotting, first identified in patients suffering from Scott syndrome (8). On the other hand, non-apoptotic transient exposure of PS to the outer membrane leaflet has been also been observed in various other cellular events, including during T cell activation (9, 10).

**Figure 1:**
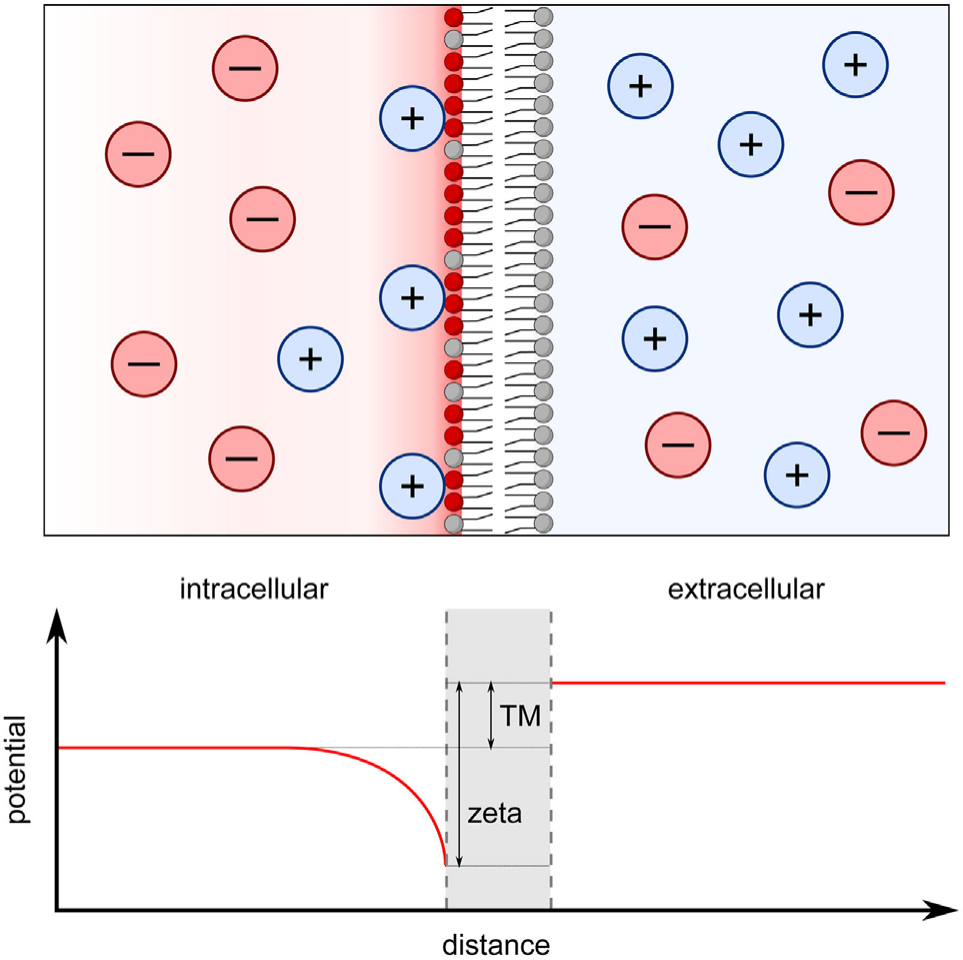
Transmembrane potential originates from both the Nernst potential and the surface charge potential. The cell interior contains higher concentrations of K^+^ and negatively charged proteins and DNA molecules while the cell exterior is more enriched in Ca^2+^ and Na^+^. K^+^ ions leak out of the cell down this concentration gradient through potassium channels leading to a net negative charge in the interior of the cell. This causes the establishment of a Nernst potential across the membrane (TM in graph), which is measured in electrophysiological patch-clamp studies. On a smaller scale, the accumulation of negatively charged phospholipids in the inner membrane leaflet generates a zeta potential with an effective range of ~ 1 nm (zeta in graph). Positively charged ions become attracted to the anionic surface, which is particularly pronounced at the inner leaflet of the plasma membrane. The surface potential at the inner leaflet is vastly different from that at the outer leaflet and the difference between the two surface potentials can be viewed as an alternative, local transmembrane potential, which can directly affect the activities of transmembrane proteins.

Another important aspect of the lipid asymmetry in the plasma membrane is the enrichment of negatively charged phospholipids in the inner leaflet. The plasma membrane is composed of ~30% PS and 0.3% PIP_2_ residing predominately in the inner leaflet, which generates a static negative surface potential of −25 mV (3, 11–13). This electrostatic potential attracts positively charged molecules from the cytoplasm and repels molecules of negative charge, as described by the Coulomb’s law (12, 14). Many peripheral membrane proteins contain positively charged motifs and can, thus, electrostatically associate with the plasma membrane because of the negative surface charge of the inner leaflet (Figure 1). For instance, it has been shown that the depletion of PS and PIP_2_ during phagocytosis causes a reduction in membrane charges at the phagosomal cup and the disassociation of polybasic membrane proteins, such as K-ras, Rac1, and c-Src (15). Similarly, the multivalent charges of PIP_2_ and PIP_3_ at physiological pH contribute to the membrane association of many polybasic-charged proteins in a synergistic manner where the depletion of either PIP_2_ or PIP_3_ alone is insufficient to cause membrane disassociation (16). Such electrostatic protein–lipid interactions can reversely modulate the effective concentration of PIP_2_ at the cytoplasmic leaflet of the plasma membrane. PIP_2_ is a source for three important cellular messengers. PIP_2_ can be hydrolyzed to diacylglycerol (DAG) and inositol 1,4,5-trisphosphate (Ins(1,4,5)P_3_), which lead to the activation of protein kinase C (PKC) pathway and intracellular Ca^2+^ release from the endoplasmic reticulum (ER), respectively. PIP_2_ can also be phosphorylated to PIP_3_, leading to the recruitment of downstream effector proteins, such as protein kinase Akt (17). A well-studied membrane lipid–protein interaction is the constitutive binding of PIP_2_ to the positively charged protein, myristoylated alanine-rich C-kinase substrate (MARCKS) (18). This interaction is proposed to act as a PIP_2_ sink that sequesters and releases PIP_2_ when associated and disassociated from the membrane (13). Detachment of MARCKS from the membrane can be triggered by elevated intracellular Ca^2+^ as the newly formed Ca^2+^/calmodulin complex is negatively charged and competes for binding to positively charged MARCKS.

## Membrane Potential: Lessons Learned from Electrophysiology

Given that the inner and outer leaflets of the plasma membrane carry predominately negatively and positively charged lipids, respectively, a transmembrane potential is created, a phenomenon that is well known in electrophysiology. Traditionally, the transmembrane potential is defined as the difference in salt ion concentrations on either side of the membrane (19, 20). Transporters, exchangers, pumps, and ion channels within the membrane, thus, maintain this transmembrane potential (21). For instance, the concentration of K^+^ ions is higher in the cytoplasm compared to the extracellular space, whereas Na^+^, Ca^2+^, and Cl^−^ ions are found at higher concentrations extracellularly (22). The higher the permeability of the ion, the stronger effect is on the transmembrane potential (19). For example, due its permeability, the resting potential of K^+^ is close to the Nernst potential, resulting in the cell interior being more negatively charged, relative to the extracellular environment (22).

Changes in the transmembrane potential have traditionally been studied using electrophysiological techniques (23). Voltage-clamp experiments provide a means to manipulate the transmembrane potential while simultaneously monitoring ion channel activity, and current-clamp mode enables measurement of membrane voltage changes that result from ion flux *via* ion channels. While powerful, such techniques do not directly interrogate the localized charge distributions at the plasma membrane nor do they capture the variations in the distribution of ions near the charged surface of the membrane (3, 14, 24–26). Conversely, the localized ion flux mediated by channel activity may lead to fluctuations in the charge distribution at the membrane. Ion channel activity can mediate a spectrum of signaling pathways as distinct families of ion channels are gated, or activated, by different signals. In addition, ion channels can exhibit selectivity for specific species of ions, resulting in either membrane depolarization (shifts toward more positive membrane potentials) or hyperpolarization. In excitable cells, these shifts in membrane potential regulate action potential generation *via* activation of voltage-gated channels. In non-excitable cells, such as T lymphocytes, the mechanism by which changes in the membrane potential regulate downstream signaling is less clear.

A number of classes of ion channels have been identified in T lymphocytes that possess diverse gating mechanisms and ion selectivity. Voltage-gated and or Ca^2+^-activated K^+^ channels function to hyperpolarize the membrane, when activated (27). The voltage-gated K^+^ channel, K_v_1.3 is gated in response to a shift from resting membrane potential to more positive potentials, thus activation of this channel directly depends on voltage changes at the plasma membrane. The electrochemical gradient of K^+^ dictates that on opening of the K^+^-selective channels, K^+^ ions will diffuse out of the cell, leading to membrane hyperpolarization (27). Such a shift toward more negative membrane potentials may have multiple downstream effects, including increasing the electrochemical driving force that promotes the influx of Ca^2+^ or Na^+^ when channels selective for these ions are activated. Depolarizing currents are thought to be mediated in T lymphocytes by TRPM4 channels, a Ca^2+^-activated, Na^+^ permeable channel (28). However, any inward flow of cations will function to depolarize the membrane. A number of Ca^2+^-conducting ion channels have been identified in T lymphocytes, including the P2X7 receptor and L-type Ca^2+^ channels (9, 29). The L-type Ca^2+^ channel belongs to the voltage-gated Ca^2+^ channel family, yet in T lymphocytes these channels are not activated by membrane depolarization (29, 30), and the precise mechanism of activation is unknown.

## Different Types of Transmembrane Potentials

As outlined above, the traditional, electrophysiological transmembrane potential of the plasma membrane is defined as the difference in electrostatic potential of diffusing ions on either side of the membrane (19, 20). This results in long-range effects, acting globally on transmembrane proteins, such as ion channels and exchangers. One can also view the transmembrane potential more locally. That is, each membrane–solution interface has its own surface potential, which is defined by the charged lipids in the membrane and the counterions in solution (Figure 1). This surface potential is often referred to as the zeta potential with a characteristic Debye length, which the distance at which is the potential decays to 1/e of its maximum (3, 14, 26). Because the inner and outer leaflets of the plasma membrane carry different charged lipids, the zeta potential at the extracellular and intracellular side also differ (24, 26). An alternative definition of the transmembrane potential is the difference in these two surface potentials (25). In this case, the asymmetrical distribution of charge lipids can affect the transmembrane potential in several ways. First, a negative zeta potential can attract positively charged ions to the membrane surface, forming an ionic double layer, as described by McLaughlin and colleagues (3, 12–14). As a result, the ionic gradient directly adjacent to the membrane may differ substantially from the gradient measured in the bulk solutions, such as in whole cell patch-clamp experiments (21, 23) (Figure 1). It is likely that channels, for example, are more sensitive to the ionic environment immediately adjacent to the membrane rather than to the distal bulk ionic concentrations. The second contribution of charged lipids to this alternative, locally defined transmembrane potential arises from interactions within the bilayer. Theoretical calculations and molecular dynamic simulations have shown that the transmembrane potential can be created solely from the difference in surface potential between the two leaflets, independently from the ionic concentration differences in the bulk solutions on either side of the membrane (25, 31). In the plasma membrane where the charged lipids are asymmetrically distributed between the two leaflets, the observed transmembrane potential can be solely described by the difference in surface potential (25). Indeed, dynamic molecular simulations have shown that a 70–100 mV transmembrane potential arises from either the asymmetric distribution of zwitterionic lipids between the two membrane leaflets or from the preferential binding of Na^+^ ions to one leaflet of the bilayer despite the ionic strength of bulk solution on either side of the bilayer being similar (32, 33). Experimentally, it has been shown that the ATP-gated cation channel P2X7 is sensitive to the translocation of PS from the inner to the outer leaflet, in other words sensitive to changes in the difference of the two surface potential but not to changes in the bulk transmembrane potential (9).

Electrostatic zeta potentials may also be directly relevant to the function of transmembrane channels and other proteins. The charged head groups of lipids create Coulomb forces that directly alter the local electrostatic environment of ion channels and, hence, can directly affect their gating mechanism (34–37). For instance, the polybasic charged motif on the cytosolic side of many ion channels, such as the voltage-gated K^+^ channels binds polyanionic PIP_2_ *via* electrostatic interactions so that the opening and closing of these ion channels are directly regulated by the local concentration of charged lipids in the inner leaflet of the plasma membrane (35, 37). In reality, it is likely that most transmembrane proteins are sensitive to both local and global electrostatic forces.

## Lateral Heterogeneities in the Membrane Give Raise to Charged Membrane Domains

It is now well recognized that the lateral distribution of membrane lipids gives rise to membrane domains. For example, the lipid raft and picket fence models propose that membrane domains are formed through lipid–lipid interactions and/or membrane interactions with the subjacent cytoskeleton (38). Recently, it has been suggested that anionic phospholipids in the inner leaflet of the membrane can also laterally assemble into nanoclusters and that this occurs in a charge-dependent manner (39–41). These membrane domains are likely to have an impact on membrane proteins. For instance, the co-clustering of lipids and proteins is responsible for the activation of the kinase K-Ras as well as activation of the so-called soluble NSF attachment protein receptor (SNARE) complexes during neurosynaptic membrane vesicle fusion. In the case of K-Ras, membrane depolarization caused by high extracellular K^+^ concentrations leads to the co-clustering of PS and K-Ras, facilitated by the electrostatic interactions between the polybasic charged motif at the C-terminus of K-Ras and the negatively charged PS lipids. These K-Ras/PS domains activate the mitogen-activated protein kinase (MAPK) signaling pathway (39). Interestingly, the amount of PS in the inner leaflet is unchanged upon membrane depolarization, highlighting the importance of local changes in the membrane zeta potential as distinct from the global transmembrane potential.

**Figure 2:**
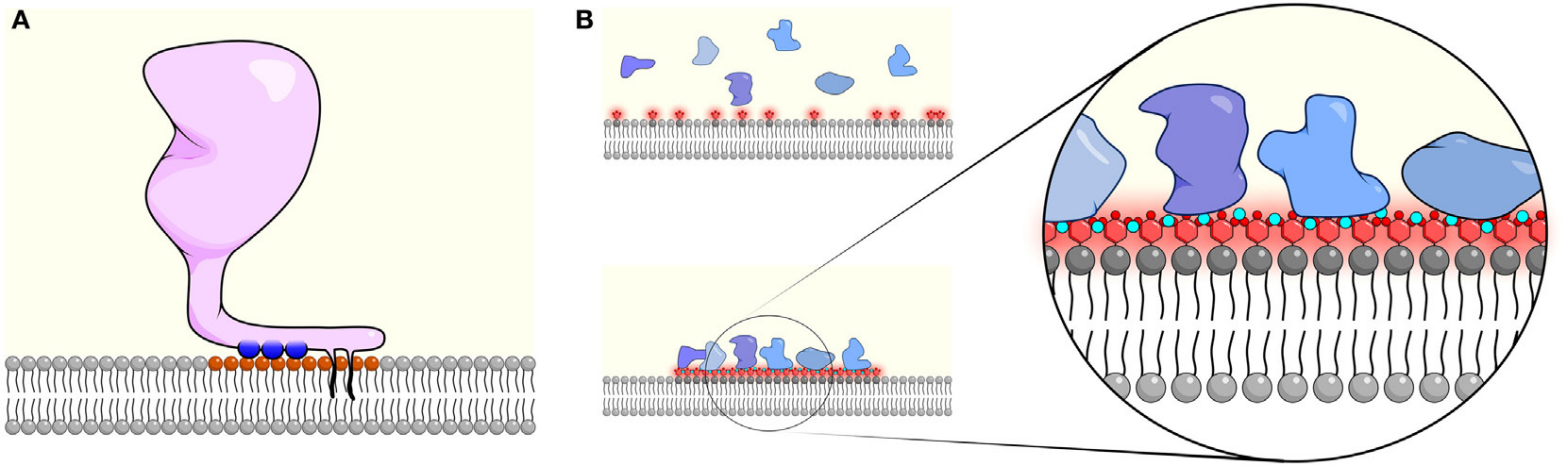
High local surface charge densities can cause membrane association and clustering of membrane proteins. **(A)** The monovalent charge of phosphatidylserine (PS) and phosphatidic acid (PA) can facilitate the membrane association of cationic molecules and can act in combination with hydrophobic interactions through palmitoylation, myristoylation, and farnesylation groups (black tails). The pairing of negatively charged lipids (orange head group) with positively charged residues (blue circles) neutralizes the membrane change and prevents additional interactions. **(B)** By contrast, multivalent interactions between phosphatidylinositol 4,5-bisphosphate (PIP_2_)/phosphatidylinositol (3,4,5)-trisphosphate (PIP_3_) (red headgroups) and Ca^2+^ (small teal circles) cause clustering of PIP_2_/PIP_3_ but do not fully neutralize the negative charge, allowing proteins containing polybasic-charged motifs (blue shapes) to be recruited. Such multivalent interactions can often lead to lateral co-clustering of PIP_2_/PIP_3_ with charged proteins, forming nanodomains at the inner leaflet of the cell membrane that can trigger activation of signaling processes.

Similar to PS, both theoretical and experimental evidence have shown that divalently charged ions such as Ca^2+^ can directly bind and laterally crosslink PIP_2_ to form PIP_2_-enriched nanodomains (Figure 2). The tetravalent charge of PIP_2_ generates strong Coulomb forces that can extend the range of the local Debye length at the inner leaflet (3, 12). As a result, PIP_2_ engages various positively charged molecules, including cations through multiple electrostatic interactions, which can lead to the lateral clustering of the protein and the lipids (Figure 2B). For instance, engaging divalent Ca^2+^ ions not only reduce the electrostatic repulsion effect of the negatively charged PIP_2_ lipids, but it can also form intramolecular and intermolecular hydrogen bonds with PIP_2_ through charge–charge interactions (42). Interestingly, although both Ca^2+^ and Mg^2+^ carry divalent, positive charges, the electrostatic interactions with PIP_2_ appear to be specific to Ca^2+^. Molecular simulation suggests that although Mg^2+^ is similarly charged, its greater hydrodynamic radius prevents it from forming strong electrostatic interactions, such as hydrogen bonds with PIP_2_ (42, 43). Many membrane and even cytosolic proteins that contain positively charged peptide motifs were found to co-localize with PIP_2_ nanoclusters (41, 44–46). One possible explanation is that the positive charge of Ca^2+^ is insufficient to neutralize the multivalent and negative charge of PIP_2_. Thus, even upon elevation of intracellular Ca^2+^ levels, Ca^2+^ and PIP_2_ nanodomains may remain highly negatively charged. As a result, proteins with multivalent, positively charged motifs would be attracted to these domains and thus form nanoclusters themselves. For instance, the polycationic SNARE protein syntaxin-1A was found to form nanoclusters with PIP_2_ when intracellular Ca^2+^ concentrations were elevated (40, 45). Such nanoclusters are responsible for the docking and fusion of the synaptic vesicles during the event of neurotransmission. Another example is the poly-lysine motif of Ebola virus VP40, which enhances PIP_2_ clustering. PIP_2_ clustering in turn is responsible for the formation of the hexamer structure of VP40 at the inner membrane leaflet, which is required for virus budding (47). In this case, the formation of membrane domains is Ca^2+^ independent but directly mediated through the electrostatic interactions between PIP_2_ and the viral protein.

## The Role of Charged Lipids in T Cell Activation

When the TCR interacts with cognate peptide presented on major histocompatibility complex (pMHC) it initiates a signaling cascade that culminates in the activation of T cells. Within the last decade it has become increasingly apparent that the local charged lipid environment around the TCR plays an important role in this process.

The TCR complex consists of alpha and beta subunits that mediate interactions with pMHC molecules, and CD3 homo- and heterodimers (CD3γ, CD3δ, CD3ε, and CD3ζ) that confer signaling potential. Upon TCR ligation, the Src family kinase, Lck, phosphorylates tyrosines within immunotyrosine-based activation motifs (ITAMs) of the CD3 chains, which become docking sites for zeta chain-associated protein kinase 70 (ZAP70). Membrane-recruited ZAP70 then phosphorylates linker for activated T cells (LAT), which recruits multiple adaptors that propagate signaling and lead to cellular effector functions such as cytokine secretion.

Basic-rich sequences (BRS) in the unstructured cytoplasmic tails of CD3ε (48) and CD3ζ subunits (49, 50), as well as the co-activatory receptor CD28 (51), cause the tails to associate with negatively charged phospholipids in the inner leaflet of the plasma membrane. NMR studies on reconstituted phospholipid bicelles composed of negatively charged phospholipids show that the tyrosine side chains of CD3ε ITAMs are buried in the hydrophobic core (48, 52). Phosphorylation of ITAM tyrosine residues prevents association of the CD3 chains with the membrane, presumably by preventing the tyrosine residues from interdigitating in the hydrophobic core and reducing the overall charge of the tail.

**Figure 3:**
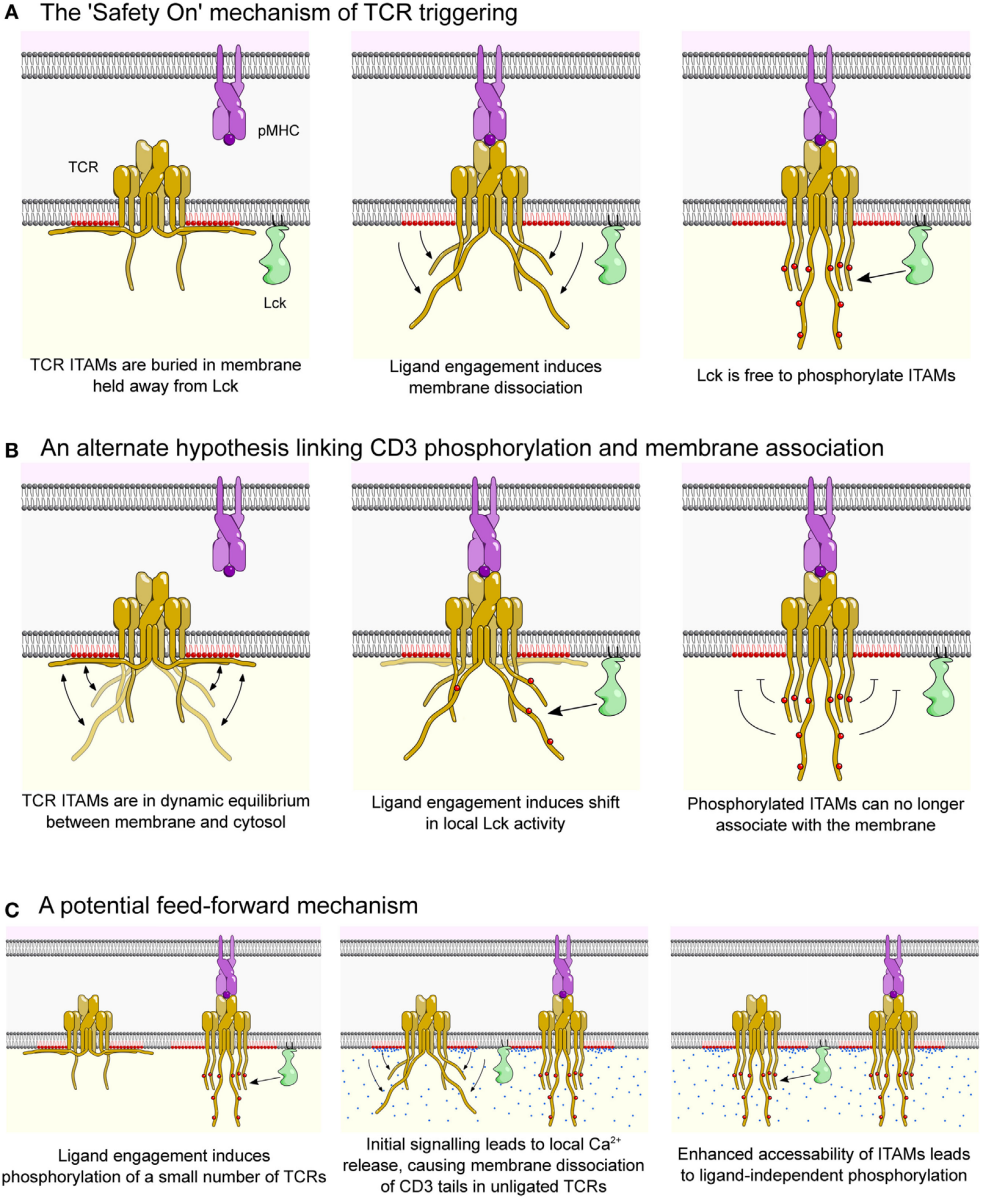
The Safety On model of the T cell receptor (TCR) triggering. **(A)** In resting T cells, the TCR complex (yellow) is prevented from spontaneously signaling by electrostatic interactions between basic-rich sequences in the cytoplasmic domains of CD3ε and CD3ζ and negatively charged phospholipids (colored red). This interaction buries critical tyrosine residues within immunotyrosine-based activation motifs (ITAMs) of CD3ε and CD3ζ in the hydrophobic core of the membrane, thus physically sequestering them from Lck (green), preventing phosphorylation and initiation of downstream signaling. When the TCR engages cognate peptide presented on major histocompatibility complex (pMHC, purple), CD3 tails are released from the membrane through an unknown mechanism, allowing them to become phosphorylated by Lck and initiate downstream signaling. **(B)** A null hypothesis for the “Safety On” model which is also consistent with current data. CD3 cytoplasmic tails are in dynamic equilibrium between being buried in and free of the membrane. Agonist pMHC-induced recruitment of Lck, and/or segregation of phosphatases [see van der Merwe and Dushek (55) for review of alternate triggering mechanisms], allows phosphorylation of CD3 chains, which prevents re-association with the membrane. **(C)** Local release of Ca^2+^ (blue circles), downstream of initial activation (first panel), may also play a role by neutralizing negatively charged lipids by releasing CD3 tails in nearby unligated TCRs and allowing them to become phosphorylated (second and third panels). This may be important for amplifying initial signaling events.

The initial observation that the cytoplasmic tail of CD3ε associates with negatively charged phospholipids led to the proposal of the “Safety On” mechanism of TCR triggering (48, 53) (Figure 3A). In this hypothesis, TCR signaling motifs are kept sequestered away from Lck until the interaction between TCR and cognate pMHC causes dissociation of CD3ε and CD3ζ tails from the membrane, allowing Lck to access and phosphorylate the ITAMs (Figure 3A). Consistent with the Safety On hypothesis, we have recently shown that lowering the electrostatic potential of the inner leaflet of the plasma membrane (through the incorporation of positively charged lipids) resulted in spontaneous phosphorylation of CD3ζ (54). Although we cannot rule out the possibility of enhanced Lck activity in these experiments, it seems likely the increased phosphorylation was a result of CD3 tails detached from membrane, which allowed access of Lck to ITAM motifs.

Two mechanistic questions arise from the “Safety On” hypothesis and remain unclear: (1) is CD3 tail dissociation a cause or consequence of phosphorylation, and (2) if tail association does block phosphorylation what is the mechanism linking TCR ligand-binding to CD3 tail dissociation?

Phosphorylated CD3ε and CD3ζ tails do not associate with the membrane, but whether full dissociation is required for, or whether it is simply a consequence of phosphorylation has yet to be proven conclusively. Recent results suggest that the association of CD3ε tails with the membrane is a dynamic process existing in multiple membrane-bound conformational states and that CD3 ITAMs are likely to be free at least a proportion of the time (52). This leaves open the possibility that CD3 tail dissociation from the membrane is not regulated by ligand engagement but is rather a consequence of ligand-mediated phosphorylation, which prevents the tail from re-associating with the inner leaflet (Figure 3B). Thus far, results of *in vitro* experiments have not definitively demonstrated that membrane association precludes phosphorylation, with poor phosphorylation of CD3ε and CD3ζ cytoplasmic domain peptides reported in the presence of negatively charged phospholipid vesicles reported by some (48, 50), whereas others observed spontaneous phosphorylation of CD3ζ in liposomes containing physiological levels of PS (56).

By contrast, results from mutation of CD3 tail BRS sequences consistently show reduced phosphorylation and loss of T cell activation (49, 57, 58). Although results from BRS mutation studies would suggest exactly the opposite of what the “Safety On” mechanism would predict, Shah et al. (59) provide a compelling alternative explanation. Their results demonstrate that Lck specifically recognizes tyrosine residues with basic-rich sequences up- and downstream, such as those in CD3ε and CD3ζ tails, whereas ZAP70 recognizes tyrosine residues flanked by negatively charged residues, such as those in LAT. Thus, mutation of the BRS to neutralize charge and reduced membrane association also likely leads to defective phosphorylation due to poor recognition by Lck, which forces a reconsideration of results based on this strategy. These results were recently supported by Li et al. (60) who showed that the BRS motif is central to the efficient binding of Lck to CD3ε, which in turn recruits the kinase to the TCR complex and allows phosphorylation of the other CD3 chains.

The cytoplasmic domains of CD3δ and CD3γ subunits lack BRS sequences, and there is no evidence that they associate with the membrane. The original “Safety On” model would predict that this could lead to the constitutive phosphorylation of ITAMs in these chains since they are exposed to Lck. The results of Shah et al. (59) and Li et al. (60) also give an explanation for why this is not the case. Although CD3δ and CD3γ cytoplasmic domains do not associate with the membrane, the lack of basic regions likely makes them poor substrates for Lck and they may require the induced proximity mediated by CD3ε–Lck interaction to become efficiently phosphorylated (60).

To summarize, it seems likely that the function of BRS sequences in the unstructured cytoplasmic tails of immunoreceptors containing immunotyrosine-based signaling motifs is twofold: firstly, to allow receptor tails to be better substrates for Lck (59), and secondly, to allow for charge-dependent association with the inner leaflet that renders phosphorylation sensitive to mechanisms regulating this association (48–50). Furthermore, rather than a binary cause and effect relationship between CD3 membrane tail association and phosphorylation, we propose that the reality lies somewhere between the extremes outlined in Figures 3A,B and that conditions used to trigger the T cell may influence how strongly phosphorylation depends on tail dissociation. For instance, conditions leading to strong phosphatase segregation and Lck/TCR colocalization may drive phosphorylation without the need to shift the equilibrium of CD3ε and CD3ζ tail association with the membrane (61). Conversely a shift in this CD3ε and CD3ζ tail equilibrium also shifts the sensitivity of the TCR to phosphorylation, to the extreme of spontaneous, ligand-independent phosphorylation in completely dissociated tails (54).

## Membrane Charges, Ca^2+^ and TCR Signaling

This still leaves the question of what regulates the interaction of CD3ε and CD3ζ tails with the membrane. Although a ligand-induced conformational change in the TCR complex has been proposed (52), convincing evidence directly linking this to tail dissociation has not been demonstrated. One mechanism that does efficiently regulate the interaction of the CD3ε and CD3ζ tails with the membrane is intracellular Ca^2+^ signaling (62). This occurs through direct association of Ca^2+^ with PS head groups, which neutralizes the charge. CD3ε and CD3ζ phosphorylation is significantly diminished, but not abolished, when cells are stimulated with anti-CD3 in the absence of Ca^2+^ or when cells are loaded with the calcium chelator BAPTA-AM (62). It should be noted that Ca^2+^ signaling is downstream of TCR activation and, thus, it is difficult to envisage how these effects could constitute the initial triggering event. Instead, any Ca^2+^-mediated effects may function as a feed-forward mechanism enhancing the sensitivity of, and/or amplifying signaling during, T cell activation (Figure 3C).

In addition to causing CD3 tail dissociation from the membrane, Ca^2+^ also causes T cell clustering (63), which may further enhance T cell activation (64). The mechanism by which Ca^2+^ causes TCR clustering is not clear at present. In our experiments, spontaneous CD3ζ phosphorylation facilitated by lowering the electrostatic interactions with the inner leaflet was not sufficient to induce TCR clustering (54), suggesting that clustering is not necessarily phosphorylation dependent. As outlined in sections above, numerous proteins containing positively charged peptide motifs co-localize with Ca^2+^/PIP_2_ nanoclusters (41, 44–46), which would suggest that this may also occur with the TCR.

A further feed-forward mechanism may come in the form of ZAP70 binding to PIP_2_/PIP_3_. The C-terminal SH2 domain of ZAP70 interacts specifically with PIP_2_ and PIP_3_ *via* a site that is distinct from the phosphotyrosine binding site of ITAMs (65). The PIP_2_/PIP_3_ interaction does not interfere with phosphotyrosine ITAM binding and appears to play an important accessory role during T cell activation leading to more robust Ca^2+^ fluxes and IL-2 production (65). Generation of PIP_2_/PIP_3_ occurs downstream of TCR signaling and the co-stimulatory receptor CD28 [reviewed in Ref. (66, 67)], and thus this mechanism may allow for prolonged membrane recruitment of ZAP70 and sustained signaling at sites of TCR/PIP_2_/PIP_3_ microclusters. Interestingly, this mechanism of stabilizing membrane interactions of proteins containing SH2 domains could apply to a wide range of signaling proteins, many of which are known to interact with PIP_2_/PIP_3_ (65). It is tempting to speculate that this is a common mechanism allowing for greater spatial specificity of SH2-containing protein recruitment to phosphorylated proteins within charged lipid microdomains.

**Figure 4:**
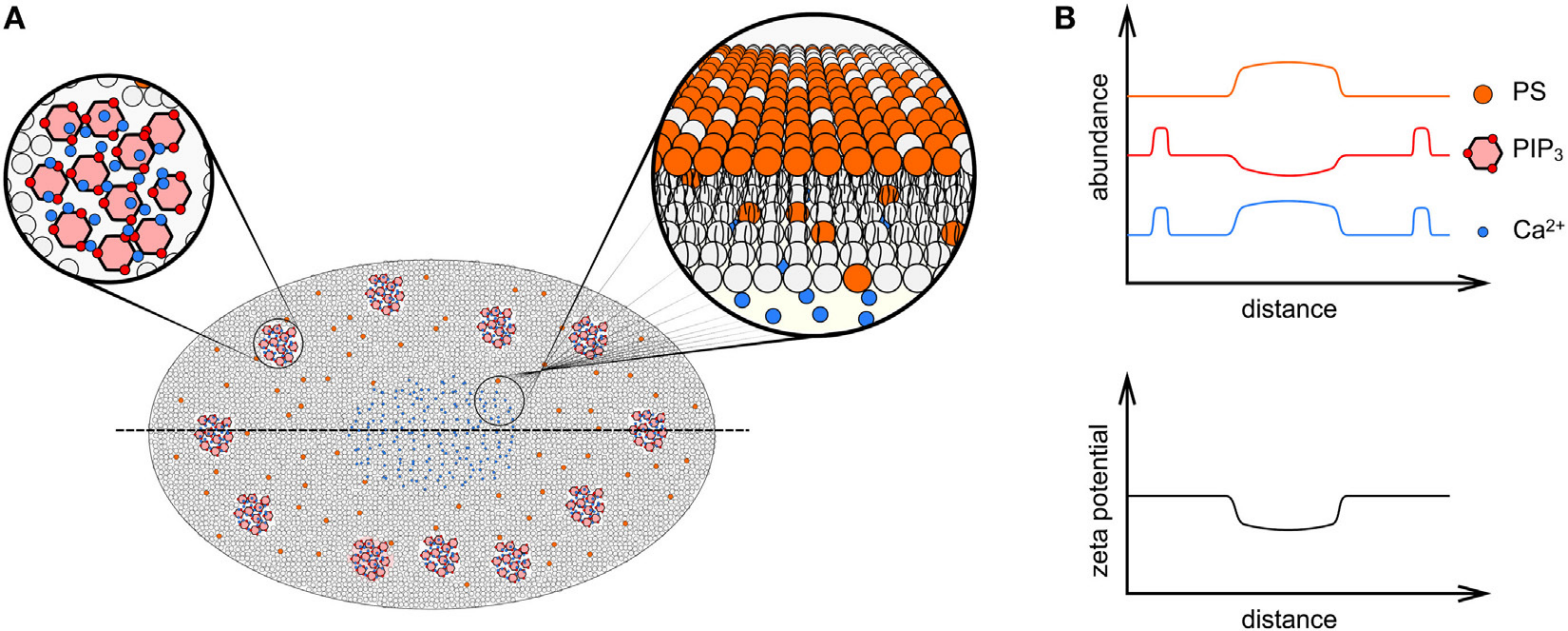
Membrane charge distribution in the immunological synapse. **(A)** An *en face* diagrammatic view of the inner leaflet of a T cell encountering a surface presenting cognate peptide presented on major histocompatibility complex ligands, showing the significant remodeling of phospholipid distribution within the mature immunological synapse. Clusters of phosphatidylinositol 4,5-bisphosphate (PIP_2_)/phosphatidylinositol (3,4,5)-trisphosphate (PIP_3_) and Ca^2+^ (red hexagons and blue circles, respectively, in top left zoomed in region) form near the periphery of the contact interface, whereas in the center of the immunological synapse PIP_2_/PIP_3_ are depleted due to the action of PLCγ. The elevated level of Ca^2+^ in the center of the immune synapse drives externalization of phosphatidylserine (PS, orange phospholipids) to the outer leaflet (illustrated in zoomed in region at top right), which are also enriched here. **(B)** Graphs of PS, PIP_2_/PIP_3_, and Ca^2+^ abundance, as well as zeta potential across line profiles through the immunological synapse [dotted line in **(A)**]. The zeta potential is lost in the center of the synapse due to externalization of PS and charge shielding by Ca^2+^.

It is now appreciated that the spatial distribution of membrane charge is not homogeneous across the immunological synapse, leading to the proposal that differently charged membrane regions are responsible for the spatial arrangement of the TCR and related signaling proteins during T cell activation (54, 68–70). For instance, Ca^2+^ influx during T cell activation occurs mainly in the center or the synapse possibly because the Ca^2+^ release-activated Ca^2+^ channels in the plasma membrane and the Ca^2+^ sensor STIM1 in the ER mostly localize to the center of the immunological synapse (71). The higher local concentration of Ca^2+^ can generate multiple effects. First, the divalent ions bind negatively charged lipids in the inner leaflet of the plasma membrane so that the effective local surface charge density is reduced (62) (Figure 4). It has been demonstrated that Ca^2+^, *via* charge screening, can directly disassociate the CD3ε tails from the inner leaflet of plasma membrane (62). Second, elevated Ca^2+^ levels can alter the activity of other enzymes that further reduce the local membrane zeta potential. This includes the activation of phospholipase Cγ (PLCγ) and suppressing the activity of membrane flippases. PLCγ hydrolyzes the polyanionic lipid PIP_2_ into DAG and IP_3_, and while the neutrally charged DAG remains at the plasma membrane, the negatively charged IP_3_ is released from the membrane and binds to IP_3_ receptor in the ER to trigger the release of Ca^2+^ from ER. As a result, the membrane surface charge at the center of the synapse is reduced. The local accumulation of DAG at the center of the synapse is responsible for the activation of members of protein kinase C (PKC) family, which leads to the recruitment of microtubule-organizing center and establishment of cell polarity and directed secretion of cytotoxic granules (70, 72).

Ca^2+^ levels also regulate the activity of flippase, which is responsible for the constitutive translocation of PS from the outer leaflet to the inner leaflet of the plasma membrane and, thus, the asymmetrical accumulation of PS at the cytoplasmic side. The membrane of the immune synapse in activated T cells is enriched with PS (73), however, a PS-specific biosensor demonstrates PS is excluded from TCR microclusters (74). The reason for this apparent discrepancy is the rise of intracellular Ca^2+^ at the center of synapse suppresses the activity of flippase so that the outward translocation of PS by floppases is dominant and overrides the inward translocation (75). As a result, PS is mostly externalized at the center and Ca^2+^-enriched regions of the synapse (Figure 4A) and the negative local zeta potential generated at the inner leaflet is reduced (74). It was also observed that CD45 is a negative regulator of PS externalization (9), which is mostly excluded from the immunological synapse due to the large size of its ectodomain (76). CD45 exclusion from the synapse center could, therefore, also contribute to the establishment of a PS gradient from center to the edge of the synapse (Figure 4B).

In contrast to the center of the synapse, the membrane charge at the peripheral regions of the synapse is much higher (54). Previous studies have shown that the multivalent, negatively charged lipid PIP_3_ was mostly located at the peripheral region of the synapse. PIP_3_ is responsible for the recruitment of Dock2 and subsequent activation of Rac1, which leads to actin polymerization and the formation of a dense actin ring surrounding the peripheral region of the synapse (69). The actin ring is required for cell adhesion and directed secretion of cytotoxic granules and cytokines (69). PIP_3_ may also be responsible for the formation of TCR clusters through multivalent electrostatic interaction with Ca^2+^ cations and polybasic charged CD3ε and CD3ζ chains of TCR complex (Figure 4A). We recently mapped the membrane charges in the immunological synapse using our Förster resonance energy transfer membrane charge sensor and showed that membrane charges were mostly homogenously distributed in resting T cells (54). Upon TCR activation, the charge was dramatically reduced in the center but maintained in the peripheral region of the synapse. Interestingly, a global reduction in membrane charges by incorporating positively charged lipids in T cells did not alter the relative charge distribution within the synapse (54). This suggests that local membrane charges within the immunological synapse are regulated separately from the global lipid composition. It is highly likely that this local regulation involves the interaction of charged lipids, ions in solution and specific T cell signaling proteins.

## Summary

In conclusion, the asymmetrical distribution of charged lipids between the two leaflets of the plasma membrane, and laterally within the leaflets, plays an important role in many cellular processes, including TCR signaling. Charged lipids in particular create an electrostatic zeta potential that not only differs on the extracellular and intracellular membrane interface but can also result in distinct membrane charge patterns, as is the case for the immunological synapse. The electrostatic potential of the inner leaflet of the plasma membrane locally regulates the transient interactions of cytosolic proteins and the association of cytosolic tails of transmembrane complexes. This is likely to control phosphorylation of the TCR–CD3 complex in T cells. Surface charges also attract ions from solution and locally restricted interactions between charged lipids with multivalent proteins and ions such as Ca^2+^ can lead to the formation of charged membrane domains or nanoclusters. Electrostatic attraction of ions to charged membranes effectively alters the ionic strength adjacent to the membrane relative to the bulk solution. This effect is sufficient to establish a transmembrane potential, which is vastly different from the one traditionally examined using electrophysiology. In addition, zeta potentials can directly control the gating of transmembrane channels, which could result in ion fluxes that in turn impact on electrostatic interactions of proteins and charged membranes. Thus, a complex and integrated picture emerges in which charged lipids, ions in solution and transient protein interactions are in a dynamic equilibrium. We are only now beginning to understand how proximal T cell signaling fits into this picture, but even with our incomplete understanding it seems clear that local, nanoscale membrane charge has important consequences for TCR function.

## Author Contributions

KG defined the scope of the review. YM and JG designed and made the figures. All authors wrote and reviewed the manuscript.

## Conflict of Interest Statement

The authors declare that the research was conducted in the absence of any commercial or financial relationships that could be construed as a potential conflict of interest.
